# Curcumin Microemulsions: Influence of Compositions on the Dermal Penetration Efficacy

**DOI:** 10.3390/pharmaceutics17030301

**Published:** 2025-02-25

**Authors:** Muzn Alkhaldi, Soma Sengupta, Cornelia M. Keck

**Affiliations:** Department of Pharmaceutics and Biopharmaceutics, Philipps-Universität Marburg, Robert-Koch-Str. 4, 35037 Marburg, Germany; muzn.alkhaldi@pharmazie.uni-marburg.de (M.A.);

**Keywords:** curcumin, microemulsion, dermal drug penetration, image analysis, ex vivo skin, passive diffusion

## Abstract

**Background/Objective**: This study provided a comparison of the influence of each component of the microemulsion formulation and investigated the impact of varying concentrations of the microemulsion components on curcumin’s ability to penetrate the skin using an ex vivo porcine ear model. **Methods**: Curcumin microemulsions with different compositions were prepared and analyzed for their physicochemical properties. The dermal penetration efficacy of curcumin was evaluated from the different formulations and compared with non-microemulsion formulations. **Results**: Findings proved that microemulsion formulations improve the dermal penetration efficacy for curcumin when compared with non-microemulsion formulations. The composition of the microemulsion affects the penetration efficacy of curcumin and increases with decreasing oil content and increasing surfactant and water content. The best penetration for curcumin is achieved with a microemulsion that contained 7.7 g of medium-chain triglycerides as the oil phase, 6.92 g of Tween^®^ 80 and 62.28 g of ethanol as the surfactant mixture, and 23.1 g water. **Conclusions**: The present study provides a foundational basis for further development of different microemulsion formulations for enhancing the dermal penetration of poorly water-soluble active compounds.

## 1. Introduction

Curcumin, a natural active compound that was found and isolated from the rhizome of the turmeric plant (*Curcuma longa* L.), is well known for its exhibiting medicinal activity to alleviate and treat numerous dermatological conditions such as acne, atopic dermatitis, and psoriasis. This can be due to its characteristics as an anti-inflammatory, anti-oxidant, and anti-bacterial agent [1,2,3,4]. Additionally, it possesses an inherent autofluorescence, a valuable characteristic that can facilitate its detection within biological tissues [5,6,7]. An important strategy to enhance the delivery and efficacy of curcumin suggests curcumin incorporation into submicron carriers such as liposomes [8], nanostructured lipid carriers [7], nanocrystals [6,9], nanoemulsions [10], and microemulsions [11,12]. Among these carriers, microemulsions, which are transparent, nanoscaled dispersions, offer several advantages, including ease of formulation and thermodynamic stability [13,14].

Microemulsions are composed of a surfactant, co-surfactant, an oil phase, and an aqueous phase. This composition enables the formation of submicron particle sizes upon dispersion, which together with their components that can act as penetration enhancers, ultimately aid in higher penetration efficacy [15,16]. Due to this, microemulsions are a favored formulation principle, especially for poorly water-soluble active compounds [2,3,4,7,17]. Various studies have shown that microemulsions enhance the bio-efficacy of curcumin [16,18,19,20], but research on the detailed information and analysis on the influence of microemulsion composition of curcumin’s penetration efficacy remains limited. In particular, insights into how variations in a curcumin microemulsion (CUR ME) composition influence particle size and, thus, the penetration efficacy of curcumin are still unclear. In other words, it is not known which kind of microemulsion composition (high oil content, low oil content, high water content, low water content, high surfactant content or low surfactant content) leads to the most efficient penetration of curcumin. The objectives of this study were, therefore, to examine the influence of microemulsion composition on the dermal penetration efficacy of curcumin. For this purpose, nine curcumin microemulsions with different amounts of oil, surfactant, co-surfactant, and water were produced (Table 1) and the dermal penetration efficacy was determined and compared to non-microemulsion formulations that contained curcumin and only one component of the microemulsion system (oil, surfactant, co-surfactant, or surfactant mixture).

## 2. Materials and Methods

### 2.1. Materials

Dry extract of *Curcuma longa* powder containing 80% curcumin was purchased from Receptura pharmacy (Cornelius-Apothekenbetriebs-OHG, Frankfurt, Germany). The oily phase used is medium-chain triglycerides (MCT), which was obtained as Miglyol^®^ 812 from Caesar & Loretz GmbH (Hilden, Germany). Polysorbate 80, commercially known as Tween^®^ 80, was from VWR International GmbH (Darmstadt, Germany). Ethanol (HPLC grade ≥ 99.8%) was acquired from Sigma-Aldrich Chemie GmbH (Steinheim, Germany). Purified water was obtained freshly in-house from a PURELAB^®^ Flex 2 (ELGA LabWater, Veolia Water Technologies Deutschland GmbH, Celle, Germany).

### 2.2. Methods

#### 2.2.1. Preparation of the Formulations

The formulations prepared in this research can be classified into two parts. Curcumin microemulsions (CUR ME) formulations and formulations of curcumin in a solution or suspension form dispersed/dissolved in one of the components of the microemulsion.

The first part of the preparation involved the production of different CUR ME formulations, following previously described methods [15,18]. The previously formulated formulations contained polysorbate 80 and ethanol as surfactant and co-surfactant, which’s concentrations were systematically changed in their concentrations in our study (Table 1). First, Tween^®^ 80 and absolute ethanol were mixed at a ratio of 10:90 (*w*/*w*) to produce the surfactant mixture (Smix). MCT oil was added in varying descending amounts, followed by the addition of different quantities of Smix to achieve different ratios of oil-Smix dispersions ranging from 90:10 to 10:90 (*w*/*w*) (Table 1). After each phase addition, the mixtures were homogenized for 3 min using a vortex shaker (IKA Vibrofix VF1 electronic, Ika-Werke GmbH & Co. Kg, Staufen im Breisgau, Germany). Purified water was then titrated drop-wise until a visually clear, translucent formulation was observed. Turbidity and cloudy or transparent appearance of the formulations were recorded during water addition. The endpoint of maximum water addition was identified by the appearance of turbidity, indicating the formation of an unstable microemulsion. Therefore, water was added in amounts just below this recorded endpoint to form the microemulsion. Finally, 0.5% (*w*/*w*) curcumin was added to the blank microemulsion formulations and subjected to sonication for 10 min using an ultra-sonicator (Elmasonic P, Elma Schmidbauer GmbH, Singen, Germany) to ensure homogeneity.

The second part of the preparation involved preparing four other formulations containing curcumin and the individual microemulsion compositions. In this step, curcumin was dissolved in either ethanol or Smix, suspended in MCT oil, or added to Tween^®^ 80. All solutions and suspensions were subjected to homogenization for 30 min using the ultra-sonicator (Elmasonic P, Elma Schmidbauer GmbH, Singen, Germany).

#### 2.2.2. Characterization of the CUR ME Formulations

##### Light Microscopy Characterization

Light microscopy (LM) was used to gain detailed information on the microstructure of the samples. Most importantly, it was utilized to visualize potential oil droplets, undissolved curcumin particles or particle agglomeration in all produced formulations. The LM images were taken using an Olympus BX53 light microscope (Olympus Corporation, Tokyo, Japan) fitted with an Olympus SC50 CMOS color camera (Olympus Soft Imaging Solutions GmbH, Münster, Germany).

##### Size Analysis and Zeta Potential Measurements

The hydrodynamic particle size diameter (z-average), polydispersity index (PdI), and zeta potential of the CUR ME formulations were determined using a Zetasizer Nano ZS (Malvern Panalytical Ltd., Malvern, Kassel, Germany). For size and PdI measurements, dynamic light scattering (DLS) was employed. For this, the samples were diluted in purified water adjusted to a temperature of 20 °C. Measurements were immediately started and performed at this temperature. This procedure was selected to avoid changes in the size of the diluted microemulsions and thermal gradients within the samples. Analysis of the results was done with the general-purpose mode, which is suggested for samples with non-monomodal size distribution [18]. For zeta potential measurements, samples were dispersed in water adjusted to a conductivity of 50 µS/cm using NaCl solution. The electrophoretic mobility (EM) was determined via laser Doppler electrophoresis and M3-PALS setup in a folded cuvette and was subsequently converted into zeta potential based on the Helmholtz–Smoluchowski equation [21].

##### pH Measurements

The pH of the prepared CUR ME formulations was measured using a pH meter (Mettler-Toledo GmbH, Schwerzenbach, Switzerland). The pH probe was immersed in the formulations, and the reading was recorded once the measurement was stable.

#### 2.2.3. Dermal Penetration Efficacy

The influence of the microemulsion composition and their ingredients on the dermal penetration efficacy of curcumin was investigated using the ex vivo porcine ear skin model after previously established protocols [22,23].

**Figure 1:**
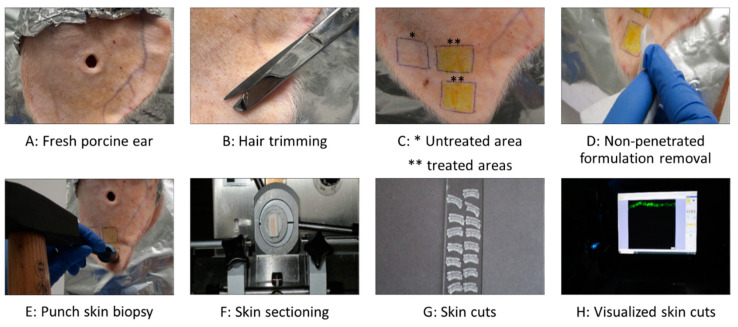
Overview of the experimental procedure of dermal penetration testing using the ex vivo porcine ear model.

The porcine ears were freshly obtained from a local slaughterhouse and utilized within hours of slaughter. In the first step, they were carefully rinsed with lukewarm water, dried using paper towels, and placed on flat polystyrene plates that were wrapped with aluminum foil (Figure 1A). Second, intact skin areas of 1.5 × 1.5 cm^2^ without visible wounds and scratches were selected and marked. Then, the hair in these areas was carefully trimmed with scissors to a length of approximately 1–3 mm (Figure 1B). Subsequently, each formulation was gently applied to the examination areas using a gloved, saturated finger, and without applying a massage (Figure 1C). The amount applied to the skin areas represented a finite dose setup (<10 µL/cm^2^) to reflect the typical condition of human exposure to chemicals [24,25], with 15 µL of the formulation applied to the 2.25 cm^2^ skin areas. Untreated skin areas served as controls. The formulations were left to be absorbed into the skin for 2 h in an oven with a temperature set to 32 °C. The short incubation time of 2 h was selected to allow curcumin penetration while simulating the practical application of such formulations containing ethanol to assess the drug penetration, particularly in formulations that are intended to treat short-term applications. Following incubation, any remaining non-penetrated formulation was gently removed using a paper towel (Figure 1D). Punch skin biopsies (Ø 15 mm) were then collected from each test area (Figure 1E). The skin biopsies were immediately embedded in Tissue-Tek^®^ (Sakura Finetek Europe B.V., Alphen aan den Rijn, The Netherlands), and then frozen and stored at −20 °C until further use. The study was conducted in triplicate using three different porcine ears, this means ears from different donors.

Due to curcumin’s strong autofluorescence [5], its dermal penetration throughout the skin can be visualized and detected using inverted epifluorescence microscopy [23]. To achieve this, the frozen punch biopsies were used to collect at least twelve of the 20 μm-thick vertical skin sections from each biopsy with a cryomicrotome (Frigocut 2700, Reichert-Junk, Nußloch, Germany) (Figure 1F). The skin sections were placed on objective slides (Figure 1G) and then subjected to inverted epifluorescence microscopy (Olympus CKX53 being equipped with an Olympus DP22 color camera, Olympus Deutschland GmbH, Hamburg, Germany). The intensity of the fluorescent light source was set to 50% and the exposure time was set to 50 ms, and the settings were kept constant throughout the analysis. The DAPI HC filter block system (excitation filter: 460–500 nm, dichroic mirror: 500 nm, emission filter: from 500 nm (LP)) was selected as the fluorescence filter for the analysis, and 40 images were obtained from each punch biopsy in this way. The experiment was performed in triplicate, which led to 120 images for each sample tested (Figure 1H). Untreated skin areas served as controls and were obtained as described above.

**Figure 2:**
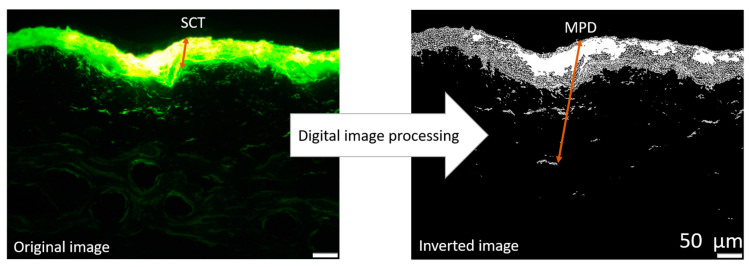
Original image: epifluorescence microscopy image of skin cut, demonstrating the scaling of stratum corneum thickness (SCT), Inverted image: original image after applying automated threshold demonstrating the measurement of mean penetration depth (MPD) of the penetrated curcumin from a formulation (200-fold magnification).

To further investigate and quantify the penetration efficacy of curcumin from the produced formulations, digital image analysis was carried out on the epifluorescence images obtained. This analysis was conducted using ImageJ software version 1.53k (National Institutes of Health, Bethesda, MD, USA) [26,27] as described in [22,23]. Briefly, to assess the penetration efficacy, all images were processed using an automated RGB-threshold protocol (cf. Supplementary Materials, Table S1) to eliminate the autofluorescence of the skin. The remaining pixels in the obtained black-and-white images (Figure 2) corresponded to the penetrated curcumin and were measured as the mean grey value per pixel (MGV/px) as a semi-quantitative measure of the amount of penetrated curcumin. In the next step, the mean penetration depth of curcumin into the skin was measured. This was done by manually measuring the distance between the upper stratum corneum and the most distant pixel in each RGB-threshold picture by using the scale function of the software, where the scale was set to 2.84 px/µm (Figure 2). In addition to the penetration efficacy and the mean penetration depth, the stratum corneum thickness was analyzed. This was done in a similar way to the mean penetration depth measurements but from the original images obtained prior to the RGB threshold (Figure 2).

#### 2.2.4. Statistical Analysis

JASP software version 0.18.3.0 (University of Amsterdam, Amsterdam, The Netherlands) [28] was used to calculate the descriptive statistics. A Shapiro–Wilk test, a widely recognized and sensitive test to assess normality, was used, while Levene’s test was applied to evaluate variance homogeneity in data that might not be perfectly normally distributed. Subsequently, parametric data were subjected to a one-way analysis of variance (ANOVA). To determine significant variations among the mean values, Games–Howell and Tukey post-hoc tests were conducted, while non-parametric data were subjected to the Kruskal–Wallis test with Dunn’s post hoc test. Statistical relationships between the physicochemical parameters and the penetration efficacy parameters (MGV/px) were also investigated by the determination of the respective correlation coefficients (Pearson’s correlation coefficient r for normally distributed data and Spearman’s rho for non-parametric data sets). Results are represented as mean values ± standard deviation (SD). *p*-values < 0.05 were considered statistically significant. Significant differences among different mean values are indicated by * *p* < 0.05, ** *p* < 0.01, and *** *p* < 0.001.

## 3. Results and Discussion

### 3.1. Production and Characterization of the Produced Formulations

Previous studies showed that Tween^®^ 80, as a biocompatible non-ionic surfactant, reduces interfacial tension, alters the lipid packing in the stratum corneum, and, therefore, enhances drug penetration into the skin. Ethanol was selected as a co-surfactant because it promotes the formation of stable microemulsion by reducing interfacial tension between the oil and water phases. Additionally, it is also used due to its ability to extract stratum corneum lipids, consequently leading to enhanced curcumin penetration [16,29,30,31,32]. MCT oil was chosen as the oil phase due to its superior curcumin solubility compared to many other oils [33,34,35]. This combination was optimized and was expected to enhance curcumin’s bioavailability by improving its solubility and delivery efficiency.

**Figure 3:**
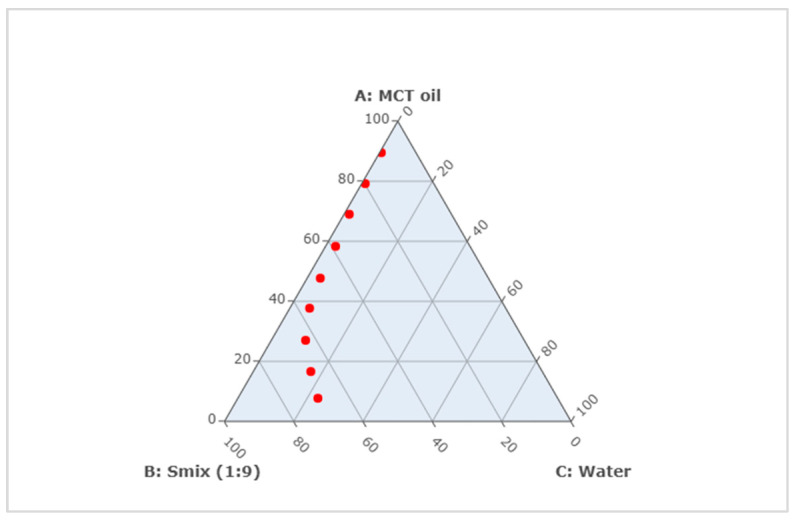
Pseudoternary phase diagram of microemulsion composed of MCT oil, surfactant mixture (Smix), and water. Red points represent the formulated CUR ME formulations (Top to bottom: CUR ME 1 to CUR ME 9).

**Figure 4:**
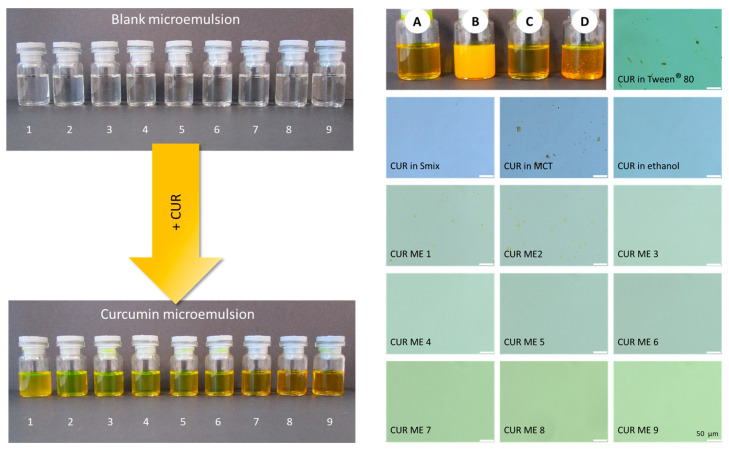
Macroscopic photos and microscopic images of the formulations. (**Left-upper**): microemulsions without curcumin, (**left-lower**): microemulsions with curcumin. (**Right-upper**): curcumin in the non-microemulsion formulations—A: CUR in ethanol, B: CUR in MCT oil, C: CUR in Smix, D: CUR in Tween^®^ 80. (**Right-lower**): light microscopic images of all produced formulations in this study (400-fold magnification, scale bar represents 50 µm).

The formulations of CUR ME with different concentrations of MCT oil, Tween^®^ 80, ethanol, and water (Table 1) were successfully prepared and plotted on the pseudoternary phase diagram (Figure 3). All formulations formed clear microemulsions (Figure 4, upper left). After the addition of curcumin to the microemulsions, a slightly turbid dispersion was observed in CUR ME 1, in contrast to the other produced CUR ME formulations, which exhibited clear yellowish solutions (Figure 4, lower left). Additionally, formulations of curcumin in the different components of the microemulsion were also produced (Figure 4, upper right: macroscopic photos A–D). Curcumin readily dissolved in ethanol and the Smix by forming clear curcumin solutions. However, it formed a suspension in the MCT oil and precipitated upon Tween^®^ 80 addition.

Light microscopy confirmed the macroscopic observations and showed undissolved curcumin particles in the oil and Tween^®^ 80 dispersion, whereas curcumin was fully dissolved in the ethanol and Smix. Larger oil droplets were seen in CUR ME 1 and also in CUR ME 2. All other microemulsion formulations were clear and contained no larger-sized droplets or curcumin particles (Figure 4, lower right).

Further characterization of the microemulsion revealed a decrease in size and PdI with decreasing oil content and increasing surfactant and water content. The zeta potential decreased with decreasing oil content and increasing surfactant and water content and the pH increased (Table 2). The PdI of all microemulsions was very high (>0.4), indicating a very broad size distribution that occurs upon the dilution of the microemulsion in water. The high PdI values upon dilution are described for microemulsions and, thus, are reasonable because it is known that the dilution process can lead to changes in the microstructure of the microemulsion that result in a broader particle size distribution [36]. The decrease in zeta potential with increasing surfactant and water content is also reasonable and can be explained by the increase in pH, which changes the charge of the ingredients and with this the zeta potential [37]. The pH values of the microemulsion formulations, ranging from 6.84 to 8.25, suggest that curcumin particles may ionize under these conditions (due to the loss of H^+^ ions from curcumin’s carboxyl groups), altering their charge characteristics [38]. In addition, the effect of the steric stabilization from the non-ionic Tween^®^ 80 [39], which forms a steric non-ionic film around the oil droplets, also influences the overall charge of the microemulsion and will decrease the charge of the droplets with increasing concentration and layer thickness. This explains why CUR ME 9, which has a higher concentration of Tween^®^ 80, shows a significantly lower negative charge than the CUR ME 1 (*p*-value < 0.001).

Overall, all CUR ME formulations formed clear microemulsions, except CUR ME 1, which showed a slight turbidity. Light microscopy confirmed undissolved curcumin in oil and Tween^®^ 80, but full dissolution in ethanol and Smix. Droplet size and zeta potential decreased with lower oil and higher surfactant/water content, while the pH increased. Tween^®^ 80 attributed to steric stabilization and reduced the zeta potential.

### 3.2. Determination of Dermal Penetration Parameters for the Formulations

**Figure 5:**
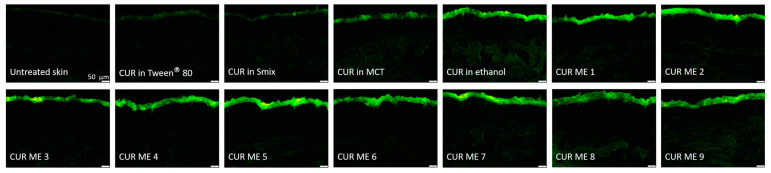
Visualization of vertical skin cuts observed through inverted epifluorescence microscopy (200-fold magnification).

**Figure 6:**
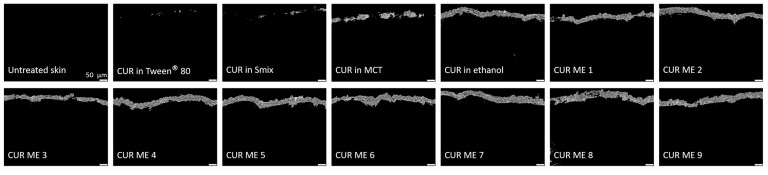
Inverted epifluorescence microscopic images after digital image processing evaluating the penetration efficacy of the formulations.

**Figure 7:**
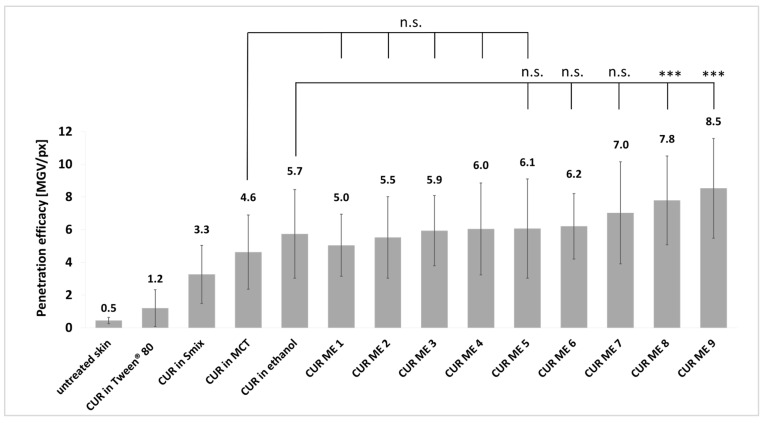
Curcumin penetration efficacies from various CUR ME formulations and non-microemulsion formulations, expressed as the penetration efficacy (penetration efficacy in MGV/px). Significant differences are indicated by asterisks (***: *p* < 0.001), while n.s. indicates no significant differences.

The epifluorescence images reveal variations in the penetration efficacy of curcumin across the different formulations (Figure 5). Generally, the microemulsion formulations of curcumin showed an enhanced penetration efficacy compared to the non-microemulsion formulations of curcumin (curcumin in oil, Tween^®^ 80, ethanol or Smix). This trend became more pronounced after digital image analysis (Figure 6), which allowed to semi-quantify the amount of penetrated curcumin into the skin from the different formulations (Figure 7).

The lowest penetration efficacy was found for CUR in Tween^®^ 80. This was expected, because the physicochemical characterization showed large curcumin particles within this formulation. As only dissolved molecules can enter the skin, this outcome was predictable. The Smix contained no curcumin particles but did not lead to a pronounced dermal penetration efficacy of curcumin from this formulation. This might be because curcumin was entrapped within the micelles. If the micelles are not able to penetrate the skin, also the entrapped curcumin will not be able to penetrate the skin. Ethanol contained the curcumin fully dissolved and the MCT oil contained the curcumin mostly dissolved with some remaining undissolved curcumin particles. Based on Fick’s law, a higher concentration gradient will lead to a more pronounced dermal penetration [40,41]. Therefore, the higher penetration of curcumin from ethanol, where all curcumin was dissolved, is reasonable. In addition, the use of ethanol as a co-surfactant might have also acted as a penetration enhancer that could further increase the dermal penetration efficacy of curcumin [42,43].

**Figure 8:**
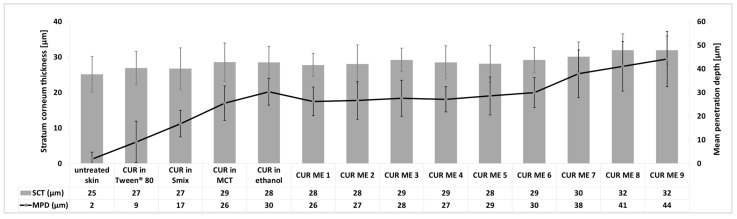
Influence of formulations of CUR ME, and solutions and suspensions of curcumin on the SCT and MPD.

CUR ME 1 to CUR ME 5 yielded similar penetration efficacies for curcumin when compared to the MCT oil and no significant differences were found between CUR ME 1–CUR ME 7 and the curcumin in ethanol. In fact, the microemulsions with high oil content and low surfactant and water content were not able to improve the dermal penetration efficacy of curcumin when compared to the non-microemulsion formulations. In contrast, the microemulsions that contained higher amounts of surfactant and water allowed for an improved dermal penetration efficacy of curcumin when compared to the non-microemulsion formulations. The highest penetration efficacy was found for CUR ME 9 which contained the highest amount of water and Smix. The dermal penetration efficacy of curcumin for the CUR ME 9 increased by 85% compared to the MCT oil formulation and was approximately 50% higher than the ethanol formulation (Figure 7). As previously discussed, the penetration enhancement effect observed with increased water and Smix concentrations can be caused due to several factors, such as the reduction of interfacial tension and altering the lipid structure of the stratum corneum, making it more permeable to active compounds. Additionally, the increase in Smix and water concentration as seen in CUR ME 9 is combined with reduced particle sizes, which further facilitates curcumin solubility and contributes to the formulation of a more stable and homogenous microemulsion that is more likely to be readily absorbed [30,32,42,43,44]. The results are also reflected by curcumin’s penetration depth from different formulations (Figure 8). However, none of the formulations were effective in transporting curcumin into the deeper skin layers. Most formulations showed a mean penetration depth less than the stratum corneum thickness, which means that curcumin penetrated into the stratum corneum but not deeper (Figure 8).

The stratum corneum thickness also reflects the effect of skin treatment on the hydration status of the stratum corneum, i.e., a higher stratum corneum thickness indicates a more hydrated stratum corneum [9,41]. The results of this study show that the stratum corneum thickness increased when the skin was treated with different formulations (Figure 8). The stratum corneum thickness was highest for the formulations with the highest amount of penetrated curcumin and the deepest penetration depth. Based on previous a study [9], this effect is caused by the presence of curcumin in the skin. Curcumin has hygroscopic properties [45] and, thus, can retain water in the stratum corneum, which then leads to an increase in the stratum corneum thickness. Consequently, higher amounts of curcumin (CUR ME 8 and 9) led to the highest stratum corneum thickness (Figure 8). The increase in the stratum corneum thickness can be also caused by the occlusive effect of the oil and/or particles [41,46,47]. However, these effects seem to be not effective in this study or were overwritten by the hygroscopic effect of curcumin in the stratum corneum.

In order to understand the influence of the microemulsion composition on the dermal penetration efficacy of curcumin in more detail, the last part of the study aimed at looking into possible correlations between the composition, the physicochemical characteristics and the dermal penetration efficacy using Spearman’s rank correlation coefficient, rho (Figure 8). A correlation coefficient value > 0.8 indicates a strong relationship between two parameters. Correlation values between 0.5 and 0.8 are considered to be moderate and rho values between 0.3 and 0.5 represent a weak correlation. Values below 0.3 are considered very weak, debatable, or as showing no correlation [48].

**Figure 9:**
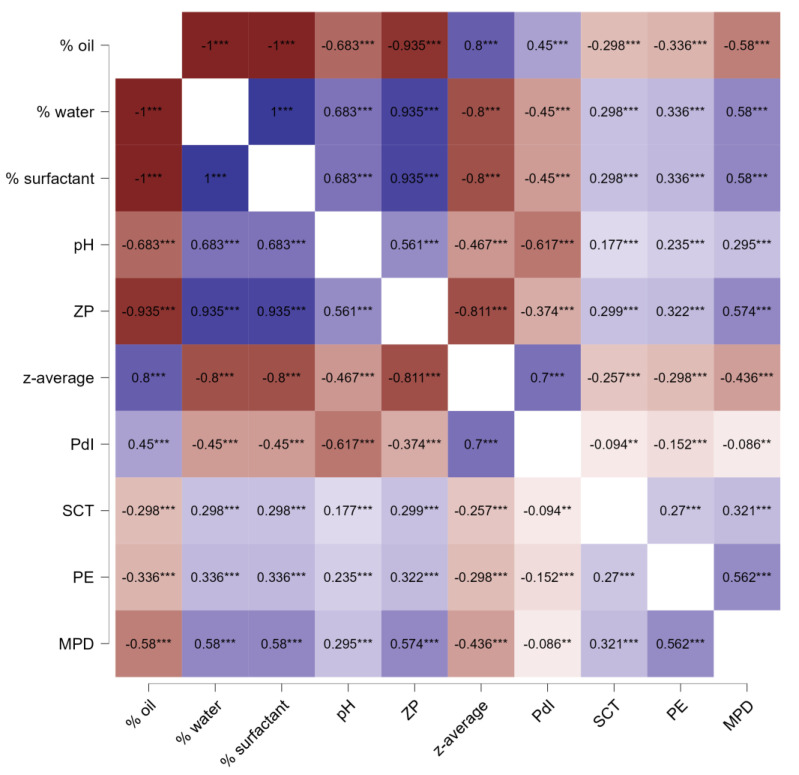
Heatmap of Spearman’s rank correlation coefficients (rho) between composition, physicochemical characteristics and penetration efficacy parameters and the physicochemical properties for all CUR ME formulations. ZP: zeta potential, PE: penetration efficacy. ** *p* < 0.01, *** *p* < 0.001.

Based on the correlation data, strong correlations were observed between the different parameters in all cases (Figure 9). The analysis revealed that a higher oil content was correlated to a larger size and a broader size distribution, while higher water and surfactant content was associated with a smaller size and narrower size distribution. This is expected, as an increased amount of surfactant combined with a reduced amount of oil that needs emulsification tends to produce smaller particles [44]. Besides, a lower oil content and a higher amount of water and surfactant were associated with a higher pH and were also associated with a lower zeta potential [38]. This correlation is also, as non-ionic surfactants provide steric stabilization [49], which means that non-ionic surfactant forms a thick layer around the droplets, leading to low zeta potential, optimally being close to zero. A larger amount of non-ionic surfactant and a lesser amount of oil that requires emulsification can be considered to yield more surfactant being available for each droplet. Hence, a thicker steric layer of non-ionic surfactant around each droplet can be formed, which then leads to a lower zeta potential, as seen in this study. Furthermore, the increased pH with increasing water and surfactant content indicates that more curcumin is dissolved and dissociated in the formulation with higher surfactant content. This is reasonable because with decreasing oil content and increasing surfactant content, the lipophilic curcumin will be more partitioned in the micelles and less in the oil phase. In the micelles, the curcumin gets in closer contact with water where it can dissociate, leading to an increase in the pH of the formulation.

The formulation parameters and physicochemical characteristics were correlated with stratum corneum thickness and penetration efficacy. Microemulsions with low oil contents, higher amounts of water and surfactant, smaller particle sizes, narrower size distribution, lower zeta potential, and higher pH values led to better skin hydration and improved dermal penetration efficacy. As a result, the best penetration for curcumin is achieved if the formulations contain high amounts of surfactants and water and low amounts of oil. In these formulations, curcumin can be considered to be more located within the micelles and not only in the oil phase. Hence, the diffusion coefficient of curcumin can be considered to increase with decreasing oil content and increasing surfactant and water content. Therefore, based on Fick’s law, the enhanced diffusion coefficient of curcumin in the formulations with low oil content but higher surfactant and water content explains the improved dermal penetration efficacy of curcumin from these formulations. The higher water content and/or the higher content of the hygroscopic curcumin in the stratum corneum explains the increased stratum corneum thickness.

## 4. Conclusions

CUR ME formulations with different compositions were successfully prepared. It was found that the size, PdI and zeta potential decreased with decreasing oil content and increasing water and surfactant content. The dermal penetration efficacy of curcumin was determined and compared to the non-microemulsion formulations. Results confirmed that microemulsion formulations are suitable formulation strategies to improve the dermal penetration efficacy of curcumin when compared to the non-microemulsion formulations. Results also indicated that the composition of the microemulsion has a strong influence on the dermal penetration efficacy of curcumin. The highest penetration for curcumin was achieved in the microemulsion formulations containing higher amounts of surfactants and lower amounts of oil.

These findings suggest that the optimized microemulsion formulations could improve curcumin delivery for potential human dermal applications, particularly for treating skin conditions such as inflammatory diseases. As curcumin is a poorly water-soluble BCS Class II or IV drug, the results of this study may provide a foundation for the development of microemulsions that enable highly efficient dermal penetration of other similar active compounds. Microemulsion, as a promising formulation, could be suitable for large-scale production, with potential applications in cosmetic products (e.g., antioxidant and anti-aging formulations) and therapeutic treatments for skin conditions like acne and eczema. However, some limitations, such as the yellowish color of curcumin, should be addressed for aesthetic purposes, and long-term stability must be evaluated for future commercial use.

## Figures and Tables

**Table 1:** Overview of the prepared curcumin microemulsion (CUR ME) formulations and their compositions, expressed as a weight percentage.

Formulation	Oil	Smix	Water	Smix Ratio	Curcumin
CUR ME 1	89.5	10.0	0.5	10:90	0.5
CUR ME 2	79.2	19.8	1.0	10:90	0.5
CUR ME 3	69.0	29.5	1.5	10:90	0.5
CUR ME 4	58.3	38.8	2.9	10:90	0.5
CUR ME 5	47.7	48.5	3.8	10:90	0.5
CUR ME 6	37.7	56.6	5.7	10:90	0.5
CUR ME 7	27.0	63.1	9.9	10:90	0.5
CUR ME 8	16.6	66.8	16.6	10:90	0.5
CUR ME 9	7.7	69.2	23.1	10:90	0.5

**Table 2:** Physiochemical characterization of the produced CUR ME formulations.

Formulation	z-Average [nm]	PdI	Zeta Potential [mV]	pH Value
CUR ME 1	5467 ± 527	0.66 ± 0.25	−7 ± 0.9	6.84
CUR ME 2	2057 ± 563	0.78 ± 0.31	−7 ± 0.7	7.33
CUR ME 3	237 ± 9	0.41 ± 0.05	−5 ± 0.6	7.85
CUR ME 4	249 ± 8	0.40 ± 0.07	−5 ± 0.4	7.81
CUR ME 5	246 ± 12	0.41 ± 0.07	−5 ± 0.2	8.35
CUR ME 6	221 ± 9	0.38 ± 0.07	−5 ± 0.05	7.86
CUR ME 7	218 ± 6	0.45 ± 0.07	−4 ± 0.03	7.74
CUR ME 8	172 ± 12	0.43 ± 0.05	−4 ± 0.05	8.12
CUR ME 9	153 ± 3	0.30 ± 0.04	−4 ± 0.03	8.25

## Data Availability

Data is contained within the article or Supplementary Materials.

## Supplementary Materials

The following supplementary information can be downloaded at: https://www.mdpi.com/article/10.3390/pharmaceutics17030301/s1, Table S1: The automated RGB threshold Macro used for the digital epifluorescence image analysis.
