# Supplementary material for: Curcumin Microemulsions: Influence of Compositions on the Dermal Penetration Efficacy
Source: Pharmaceutics. 2025 Feb 25;17(3):301. doi: 10.3390/pharmaceutics17030301 (PMC11944443; doi:10.3390/pharmaceutics17030301)
Supplement: Supplementary file 1 [file pharmaceutics-17-00301-s001.zip › pharmaceutics-3465635-supplementary.pdf]

pharmaceutics

Supplementary material

Curcumin microemulsion: influence of compositions on the dermal penetration efficacy

Muzn Alkhaldi, Soma Sengupta and Cornelia M. Keck *

Department of Pharmaceutics and Biopharmaceutics, Philipps-Universität Marburg, Robert-Koch-Str. 4, 35037 Marburg, Germany; muzn.alkhaldi@pharmazie.uni-marburg.de (M.A.); masogh@gmail.com (S.S.)
* Correspondence: cornelia.keck@pharmazie.uni-marburg.de (C.M.K.)

Table 1: The following ImageJ macro was recorded and used to subtract the autoflorescence of the skin for the images obtained from inverted epifluorescence microscopy:

```
// Color Thresholder 1.53k
// Autogenerated macro, single images only!
min=newArray(3);
max=newArray(3);
filter=newArray(3);
a=getTitle();
run("RGB Stack");
run("Convert Stack to Images");
selectWindow("Red");
rename("0");
selectWindow("Green");
rename("1");
selectWindow("Blue");
rename("2");
min[0]=0;
max[0]=0;
filter[0]="stop";
min[1]=66;
max[1]=255;
filter[1]="pass";
min[2]=0;
max[2]=0;
filter[2]="stop";
for (i=0;i<3;i++){
  selectWindow(""+i);
  setThreshold(min[i], max[i]);
  run("Convert to Mask");
  if (filter[i]=="stop")    run("Invert");
}
imageCalculator("AND create", "0","1");
imageCalculator("AND create", "Result of 0","2");
for (i=0;i<3;i++){
  selectWindow(""+i);
  close();
}
selectWindow("Result of 0");
close();
selectWindow("Result of Result of 0");
rename(a);
// Colour Thresholding-------------
run("Invert");
```
